# Signaling Pathways Associated with Chronic Wound Progression: A Systems Biology Approach

**DOI:** 10.3390/antiox11081506

**Published:** 2022-07-31

**Authors:** Proma Basu, Manuela Martins-Green

**Affiliations:** Department of Molecular, Cell and Systems Biology, University of California Riverside, Riverside, CA 92521, USA; promab@ucr.edu

**Keywords:** WGCNA, gene–trait correlation, diabetic model, RNASeq, STRING, wound healing

## Abstract

Previously we have shown that several oxidative stress-driven pathways in cutaneous chronic wounds are dysregulated in the first 48 h post-wounding. Here, we performed an RNASeq analysis of tissues collected up to day 20 after wounding, when we have determined full chronicity is established. Weighted Gene Correlation Network Analysis was performed in R segregating the genes into 14 modules. Genes in the modules significantly correlated (*p* < 0.05) to early and full chronicity were used for pathway analysis using pathfindR. In early chronicity, we observed enrichment of several pathways. Dysregulation of Ephrin/Eph signaling leads to growth cone collapse and impairs neuronal regeneration. Adra2b and Adra2a overexpression in early and full chronicity, respectively, decreased cAMP production and impaired re-epithelialization and granulation tissue formation. Several pathways involving a Smooth-muscle-actin (Acta1) were also enriched with Acta1 overexpression contributing to impaired angiogenesis. During full chronicity, the ‘JAK-STAT’ pathway was suppressed undermining host defenses against infection. Wnt signaling was also suppressed, impairing re-epithelialization and granulation tissue formation. Biomarkers of cancer such as overexpression of SDC1 and constitutive activation of ErbB2/HER2 were also identified. In conclusion, we show that during progression to full chronicity, numerous signaling pathways are dysregulated, including some related to carcinogenesis, suggesting that chronic wounds behave much like cancer. Experimental verification in vivo could identify candidates for treatment of chronic wounds.

## 1. Introduction

Chronic wounds develop because of defective regulation of the complex molecular and cellular processes involved in proper healing and have a significant impact in health care [1,2,3]. The cost of treating chronic wounds is between $28 to $31 billion annually among Medicare recipients and affects nearly 8.2 million people (https://www.woundcarestakeholders.org/news/studies-and-publications/chronic-wounds-economic-impact-costs-to-medicare, accessed on: 16 June 2022) [4]. Although several studies have been performed to address processes involved in chronic wound development and progression, to date the scientific community in wound healing has been unable to crack the very difficult, complex, and multi-dimensional processes involved in chronic wound development.

Wound-healing consists of different phases, starting with homeostasis after the initial injury followed by an inflammatory phase, a proliferation phase, and then the final phase, remodeling. Chronic wounds have increased oxidative stress (OS) from the early stages after injury, due in part to insufficient antioxidant molecules to remove the oxidative radicals. In our previous studies describing the development of the model we determined that superoxide dismutase (SOD,) an enzyme that produces H_2_O_2_, is significantly elevated, and consequently, H_2_O_2_ is also elevated shortly after injury. Moreover, we also found that Catalase is decreased whereas Gpx is not increased, indicating that OS is high [2]. Moreover, in a more recent publication discussing initiation of chronic wound development, we observed that the PI3k/Akt signaling pathway is downregulated as early as 6 h post wounding, which leads to lack of activation of NRF2 mediated antioxidant pathways. Therefore, reactive oxygen species are not scavenged resulting in tissue damage [5]. In addition, these wounds have chronic inflammation, impaired re-epithelialization to close the wound, and abnormal dermal–epidermal connectivity that results in poor dermal epidermal interaction, damaged microvasculature, and abnormal collagen matrix deposition in the wound tissue [6,7]. In addition, they contain biofilm-forming bacteria [8,9,10].

During the inflammatory phase, chronic wounds show increased numbers of all three types of leukocytes, neutrophils, macrophages, and lymphocytes found in the wound bed simultaneously. Normally, neutrophils come to the wound site a few hours after wounding, then macrophages replace them, and finally, toward the remodeling phase, lymphocytes invade the wound tissue [11]. The fact that these three types of leukocytes are all present at the same time in the very early stages of chronic wounds indicates that inflammation in chronic wounds is also confused, in addition to being chronic. However, it should be noted that neutrophils may not always be present in chronic wounds.

Neutrophils secrete myeloperoxidase that in turn use H_2_O_2_ to produce various types of hypohalous acids [12,13]. Therefore, increased amounts of myeloperoxidase in chronic wounds relate to larger amounts of hypohalous acids, which can be caustic and damaging to the already fragile wound tissue. Neutrophils also participate in killing of microbes in the wound [14]. Other inflammatory mediators such as arachidonic acid and its metabolites are also secreted during this phase and enhance the inflammatory response and cause pain. 

In normal wound-healing, the proliferative phase lasts from a few days to a few weeks. This phase is marked by the proliferation and migration of keratinocytes at the wound edge, resulting in wound closure [15,16]. Fibroblasts and myofibroblasts also begin to migrate from the wound edge, and the pro-inflammatory macrophage population is replaced with anti-inflammatory macrophages. In chronic wounds, various factors such as secreted catecholamines prevent the proliferation and migration of keratinocytes reducing the rate of wound closure. The alternative activation of anti-inflammatory macrophages is also inhibited in chronic wounds prolonging the presence of pro-inflammatory macrophages in the wound [17]. Processes related to energy metabolism and ATP production are also disrupted in chronic wounds, which together contribute to the formation of a dysfunctional wound tissue unable to repair itself. The final phase of healing, remodeling, is marked by remodeling of the granulation tissue to produce scar. This phase does not occur in chronic wounds; the wound tissue does not form.

Little is known about the signaling pathways and gene expression patterns that are involved in the progression of wounds to chronicity. In this study we examine the significantly correlated genes and the significantly enriched pathways that lead to chronic wound development and progression using Weighted Gene Correlation Network Analysis (WGCNA). WGCNA divides all the identified genes given as input, into various modules according to correlation based on the expression of the genes. Modules are chosen based on the concept of scale-free topology, which means that the characteristics of the network are independent of the size of the network. The size of the network is measured in the number of input genes also called nodes. However, irrespective of the number of nodes the basic structure of the network remains the same. Networks that follow scale-free topology but are randomized in nature for all other properties are best suited for biological systems because we can see the network as a probability [18,19]. An important consideration while comparing experimental and control conditions in biological systems is reduction of background noise. In scale-free networks, a soft power threshold is necessary for such noise filtering.

In visualizing biological data as a network, each gene or protein serves as a node and the data correlating two nodes, for example, co-expression, evidence of interaction, serves as the edge that connects the node and defines the connectivity of the node. The more connected a node is to other participating nodes in the network, the bigger the importance of that node which then becomes a HUB gene. In order to make sure that the connectivity and thereby the importance of the node is relevant to the comparison being addressed in the experiment, selecting an appropriate power for network construction is necessary. To obtain a network with scale-free topology having a co-efficient of correlation greater than 0.8, a power less than 30 is appropriate for signed networks. In our analysis of gene expression data discussed in this paper a signed network is used so that both positive and negative correlation of genes to experimental conditions, can be evaluated [20,21].

In this study, we found that distinct signature pathways are activated in chronic wounds which create a microenvironment leading to non-healing. We discuss these pathways in terms of early chronicity during the development of chronicity, which ranges from Day 1 to Day 10 post-wounding in our mouse model of chronic wounds and full chronicity at Day 20 post-wounding. Our mouse model develops chronic wounds because of high oxidative stress present in the wound tissue due to reactive oxygen and nitrogen species that are not scavenged due to the decrease of antioxidant molecules and enzymes. Our previously published studies also show that treatment of the wounds with externally applied antioxidants such as NAC and α-tocopherol reverse chronicity. Therefore, the relation between development of chronic wounds and oxidative stress is well established. Here we describe the changes in gene expression resulting from prolonged oxidative stress in chronic wounds. We discuss the significantly identified pathways for early and full chronicity highlighting the genes that act as master regulators for the dysregulation of the pathway. Our rationale for doing this study was to identify the transcriptomic variations that establish chronicity in wounds and find key genes that are dysregulated in chronic wounds to select targets for effective treatment of chronic wounds.

## 2. Materials and Methods

All experiments were completed in accordance and compliance with federal regulations and the University of California policy and procedures approved by the UCR IACUC (Institutional Animal Care and Use Committee) ethical code 20200024 (approval date 30 July 2020 and expires 29 July 2023).

**2.1 Sample Collection**: The db/db^−/−^ mouse model has a knockout mutation in the leptin receptor of the C57BLKS/J background that makes them diabetic and obese. The db/db^−/−^ mice were only used for wounding once they were 5 months old and weigh ≥ 50 g. The mice needed to be this old to be fully diabetic and obese and needed weight > 50 g in order to withstand the burden of wound chronicity. For the creation of chronic wounds, 7 mm diameter full-thickness cutaneous wounds were made, and a one-time treatment with inhibitors of catalase and glutathione peroxidase (GPx) was performed [22,23]. Briefly, twenty minutes prior to wounding, mice were treated once intraperitoneally (IP) with 3-amino-1,2,4-triazole (ATZ) (Aldrich Chemistry; St. Louis, MO, USA) at 1 g/kg body weight, an inhibitor for catalase. Immediately after wounding, they were treated once topically with the inhibitor for GPx, mercaptosuccinic acid (MSA), (Sigma Lifesciences; St. Louis, MO, USA) at 150 mg/kg body weight. Immediately after wounding, the wounds were covered with tegaderm (3 M; St. Paul, MN, USA) to prevent contamination. The wounds were kept covered for the duration of the experiments. In db/db^−/−^ mice, hair grows back very slowly after shaving; hence the tegaderm remained in place [22]. Control wounds were treated with the vehicle (PBS) instead of catalase and GPx inhibitors and remained non-chronic. Non-chronic wounds in diabetic mice heal around day 20, whereas chronic wounds remain open for weeks and sometimes months if the mouse survives. At time of wound collection, the mice are euthanized with carbon dioxide. Up to 3 mm away from the wound, margin was excised and immediately frozen in liquid N_2_. Samples were stored at −80 °C for subsequent RNA extraction. Two biological replicates each were collected for chronic and non-chronic wounds at days 1, 5, and 10 post-wounding, two replicates each of db/db^−/−^ skin and chronic wounds, 20 days post-wounding, were also collected.

**2.2 mRNA Extraction and RNASeq analysis:** mRNA was extracted with and purified using the RNeasy Plus Mini Kit, Cat no. 74134. The mRNA was sequenced with Illumina Hi-Seq (Illumina, Inc., San Diego, CA, USA) with single-end 50 base at UC Riverside Institute for Integrative Genome Biology (IIGB). After the mRNA was sequenced, the FastQC reports were checked for quality. All samples recorded reads with Phred score > 30, which confirmed the quality of reads at 99.9% accuracy, before analysis. Programs available at command line were used to analyze the reads. The fastq files were then aligned to a recent mouse genome assembly (GENCODE Release M25, Mus_musculus. GRCm38.p6) with hisat2 (2.2.1). Kallisto [24] was used to align and estimate read counts from fastq files (Table S1). The raw read counts were then obtained and normalized to Transcripts Per Million (TPM) using R (4.0.3) (Table S2). The annotation file from the Gencode Release M25 was used in the R analysis to identify exons and summarize read counts at the gene level. The analysis identified 21,919 genes altogether from 15 samples.

**2.3 Weighted Gene Correlation Network Analysis (WGCNA):** WGCNA is a comprehensive method of identifying correlation networks. The RNASeq analysis identified, 21,919 genes that showed expression across all samples. The TPM normalized expression dataset was not filtered by differential expression. Using the package WGCNA in R and analyzing scale-free topology with a soft threshold value of 14, an adjacency matrix was calculated using the Pearson correlation method. The adjacency matrix was then used to identify modules according to the correlated co-expression of the genes. The constituent genes of each module were assigned a ‘module membership’ correlation value that gauged if the gene truly belonged to the module (Pearson correlation, *p* < 0.05). These modules were then correlated to the experimental conditions (dichotomous matrix of whether the wound tissue was collected from chronic or non-chronic wound) via the module eigengenes. Module eigengenes represent the gene expression profile of each module. The module eigengenes that correlated to each timepoint in chronic and non-chronic wounds were then checked for significance (Pearson correlation, *p* < 0.05). Gene trait significance (Pearson’s correlation method) was calculated for the genes in the significant modules and only genes that showed both significant module membership (*p* < 0.05) and correlation to traits (*p* < 0.05) were used for further analysis.

**2.4 Pathway analysis for significant modules identified by WGCNA:** For the discussion of the broader transcriptomic landscape at each timepoint, all the genes from the significantly correlated module were used as input for the R package pathfindR (version 1.6.2) to identify significantly enriched pathways [25]. The protein–protein interaction database for *Mus musculus* from STRING was used to identify enriched signaling pathways and to visualize network patterns and interactions between genes. The pathfindR package identified significantly enriched pathways which were precise and mapped out the progression of wound through the timepoints. Only pathways with FDR < 0.05 were reported in the Results section.

## 3. Results

**Figure 1:**
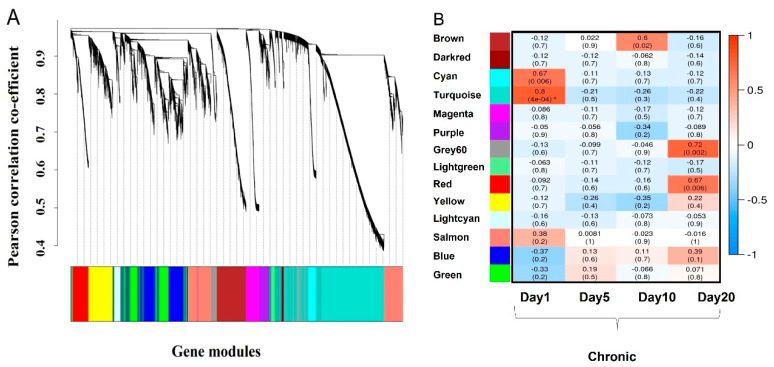
Dendrogram of all genes identified by RNASeq by hierarchical clustering. (**A**) The genes identified in the RNASeq were clustered into modules using WGCNA. The modules were named after different colors which are plotted on the *X*-axis. Hierarchical clustering was used for clustering the genes into modules according to correlation. The height of the gene tree plotted on *Y*-axis is a measure of the Pearson correlation co-efficient. (**B**) The module trait heatmap plots the timepoint (days) after wounding on the *X*-axis and the module eigengene. Eigengenes are pseudogenes representative of the expression value of genes included in the respective module of the identified modules from RNASeq on the *Y*-axis. The value in the boxes is the Pearson correlation co-efficient of the module eigengene to the respective timepoint on the *X*-axis and the value in the bracket is the statistical significance (*p*-value) for the correlation. Note: * the p-value of the turquoise module on Day1 is 0.0004.

For this study, RNASeq was performed during early chronicity (first 10 days post-wounding) and at full chronicity (20 days post-wounding) to provide an overview of the progression of the wound to chronicity, so that we can compare progression to healing in acute wounds to failure to healing in chronic wounds. We discuss our analysis of the RNASeq data using the Weighted Gene Correlation Network Analysis (WGCNA) (Figure 1A). During the first 10 days post-wounding, chronic wounds were still developing. We observed various pathways significantly correlated at Day 1 post-wounding, but by Day 5, the wound entered a state of stasis and appeared to be paralyzed in terms of transcriptional changes. The chronic wound transcriptome changed again starting Day 10 and continued to change up until Day 20, when the wound was fully chronic.

### 3.1. Signaling Pathways Present in Early Chronicity

When the RNAseq data was analyzed by WGCNA, we found that three modules were significantly correlated to early chronicity: the cyan (0.67, *p* = 0.006), the turquoise (0.8, *p* = 0.0004, and the brown (0.6, *p* = 0.02) modules (Figure 1B). The cyan module contained 346 genes (Table S3), the turquoise module 2920 genes (Table S4), and the brown module consisted of 619 genes (Table S5), all of which showed significant module membership and gene trait significance (*p* < 0.05).

**Figure 2:**
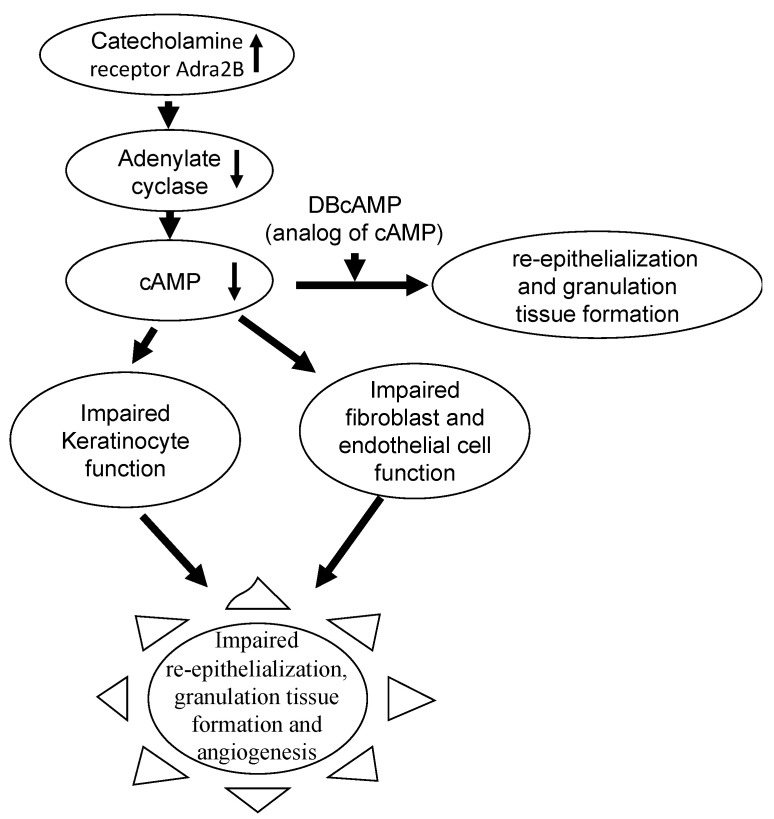
Overexpression of Adra2B in early chronicity. The increase in catecholamine producing enzymes is observed during initiation of chronicity as early as 12 h after wounding and has been reported in Basu et al., 2021. During progression Adra2B, a receptor of catecholamines, is also overexpressed. This leads to downregulation of adenylate cyclase and consequently of production of cAMP, a second messenger crucial for keratinocyte proliferation and migration, fibroblast function and angiogenesis. This contributes to lack of re-epithelialization in chronic wounds.

***A.1. The significantly enriched pathway identified for the cyan module is***: ‘Neuroactive ligand-receptor interaction’ pathway which showed significant fold enrichment (Table 1). The ‘neuroactive ligand-receptor interaction’ in the cyan module consists of several genes, the most significant of which was Adra2b (adrenergic receptor, alpha 2b), which is overexpressed in chronic wounds (3.47-log_2_ fold). The ADRA2B receptor mediates catecholamine-induced inhibition of adenylate cyclase which is the enzyme responsible for producing cAMP, an important second messenger for G-protein coupled receptors. The ADRA2B-mediated inhibition of adenylate cyclase causes decreased production of cAMP which decreases signal transduction by G-protein coupled receptors present in keratinocytes inhibiting re-epithelialization (Figure 2).

Wounds in 8–12-week-old *db/db^−/−^* mice that were normally impaired showed significant improvement in re-epithelialization and granulation tissue formation after application of DBcAMP, an analog of cAMP (Figure 2) [26].

In fibroblasts, the lack of cAMP affects the production of cytokines, chemokines, and growth factors such as IL-6, IL-8 and TGFβ, impairing fibroblast proliferation/migration and also angiogenesis. The latter processes are critical for proper granulation tissue formation (Figure 2) [27].

***A.2. The significantly enriched pathways identified in the turquoise module are***: ‘Axon guidance’, ‘Regulation of actin cytoskeleton’, ‘ErbB signaling pathway’, ‘Focal adhesion’ and ‘Proteoglycans in cancer’ (Table 1).

‘Axon guidance’, ‘Regulation of actin cytoskeleton’: the enrichment of ‘Axon guidance’ and ‘Regulation of actin cytoskeleton’ can be discussed together as both pathways are required for neuronal growth and axon movement and are orchestrated by the Ephrin/Eph (Ephrin ligand/Eph receptor)-mediated signaling pathways [28,29]. Following injury, the growth cones in neurons respond to various chemotropic cues, which can be attractive and promote the directional movement of the axon, or repulsive cues that cause repulsion and growth cone collapse [29].

**Figure 3:**
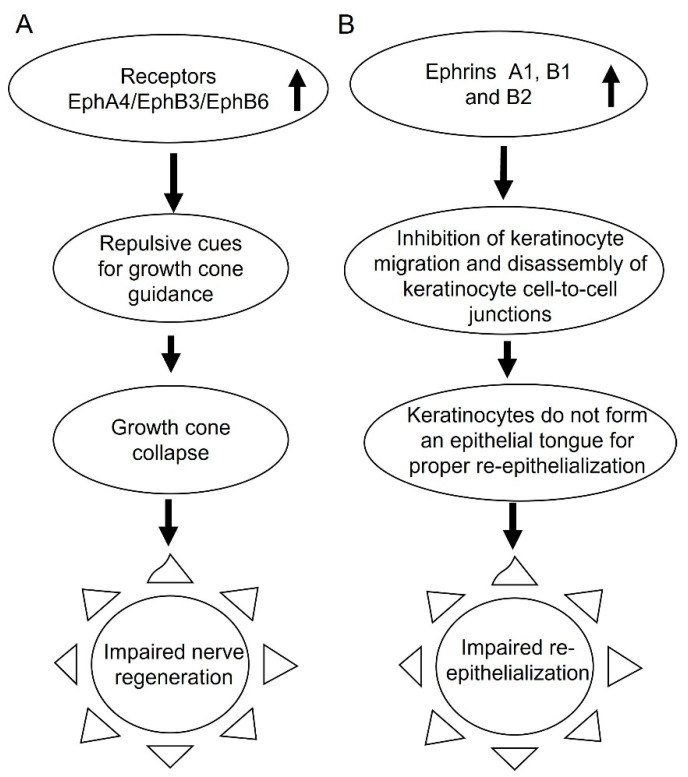
Ephrin signaling is dysregulated during early chronicity. Ephrin signaling has many repercussions when dysregulated. (**A**) Overexpression of Eph receptor A4, B3, and B6 results in repulsive cues leading to growth cone collapse. This hinders healing of motor and sensory neurons after injury in chronic wounds and causes impaired nerve regeneration. (**B**) Overexpression of Ephrin A1, B1, and B2 inhibits keratinocyte migration and causes dissolution of cell-to-cell junctions. This results in individual keratinocyte cells not forming an epithelial tong and wandering into the wound bed failing to re-epithelialize.

In our data Ephrins A1, A3, A4, A5, and receptors EphA1, A2, A4, A7 are overexpressed during very early chronicity (Table 2). In rodents, upregulation of EphA4 following spinal cord injury prevented recovery by inhibiting the movement of axons across the injury site in spinal cord injuries [28,30]. In our data EphA4 is 3.12-fold overexpressed thereby suggesting that growth cone regeneration does not occur in chronic wounds. Moreover, in very early chronicity, EphB3 and EphB6 are also overexpressed by 2.22 and 3.33 log fold change, respectively (Table 3). The signaling by these receptors modulates repulsive cues towards growth cone guidance and is associated with growth cone collapse that impair motor nerve regeneration, which in turn adversely affects sensory nerve regenerations [29] (Figure 3A).

Another repulsive cue for growth cones is the overexpression of Robo2 in chronic wounds. ROBO 1–4 are single pass transmembrane receptors and interact with Slits which are present in the extracellular matrix. In *Drosophila*, Robo–Slit interactions lead to growth cone repulsion [31]. *Drosophila* ROBO2 has two sequences in the cytoplasmic domain in common with mammals, CC0 and CC1. During embryonic development in *Drosophila*, low levels of Robo2 results in too many axons to cross the midline, whereas overexpression of Robo2 repels axons from crossing the midline. In addition, at higher levels Robo2 also repulses axons away from Slit, where Slit is secreted by the midline and acts as a repellent. In chronic wounds, Robo1 (2.52 log fold), Robo2 (4.35 log fold) and Slit 1 (1.94 log fold) are overexpressed, thereby indicating an abundance of repulsive cues to the growth cone progression leading to decrease of neuronal activity in chronic wounds. The Eph/Ephrin pathways and the Robo/Slit pathways converge during early neurogenesis [32].

Proper regulation of the Ephrin/Eph signaling pathways is also required for re-epithelialization and blood vessel morphogenesis, in addition to axon guidance [33,34,35]. Upon activation of EphA1 by Ephrin A1, cell attachment to the extracellular matrix occurs, inhibiting cell spreading and motility [36]. The overexpression of both EphA1 and Ephrin A1 in early chronicity suggests increased attachment of keratinocytes, to the extracellular matrix inhibiting their motility and hence contributing to the inability of keratinocytes to migrate and form the epithelial tongue over the wound bed, a process that is critical for proper closure of the wound (Figure 3B).

It has also been shown that EphrinB-mediated signaling is essential for release of cell-to-cell adhesion junctions. In normal healing this allows for cell migration and thereby facilitates re-epithelialization [37]. However, these investigators also observed that overexpression of EphrinB1 and B2 leads to individual epithelial cells detaching at the leading edge of the wound therefore, instead of forming a re-epithelialization tongue the detachment of individual epithelial cells impairs re-epithelialization of the wound. Because our data show that both EphrinB1 and B2 are overexpressed in early chronicity, this could contribute to the lack of re-epithelialization in our mouse chronic wounds (Figure 3B).

**Figure 4:**
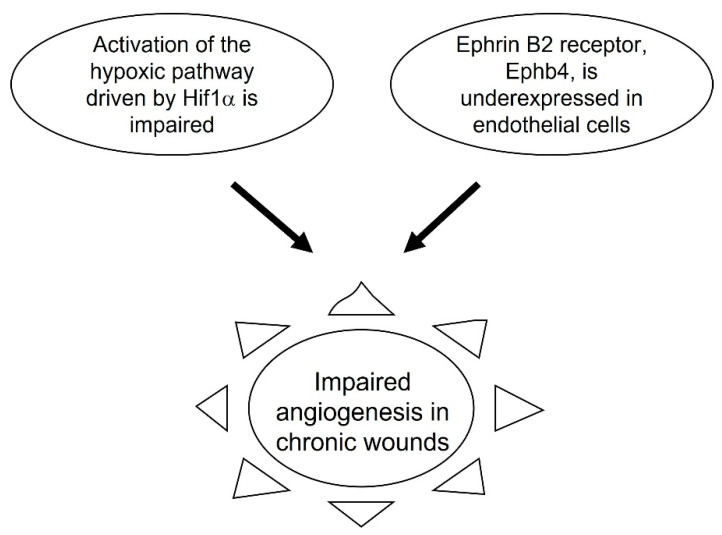
Impaired angiogenesis during progression to chronicity. Angiogenesis is compromised in chronic wounds. Hypoxia signaling is required for angiogenesis to occurs. However, hypoxia signaling via Hif1a in diabetic patients is impaired, and this problem becomes more aggravated during chronic wound development. Expression of Ephrin B2 in endothelial cells is also important for angiogenesis. In chronic wounds, Ephb4, the receptor for Ephrin B2, is under-expressed, resulting in impaired angiogenesis.

The overexpression of the receptors for EphrinB in early chronicity also impairs vascularization. Specifically, Ephrin B2 is required for angiogenesis. The process of angiogenesis is driven by hypoxia. However, in diabetic patients, hypoxia signaling is impaired and the activation of the hypoxic pathway via Hif1α is impaired. Under normal conditions when hypoxia is activated by Hif1α, there is also an increase in expression of Ephrin B2 in endothelial cells [38]. In chronic wounds however, not only is the hypoxic signaling impaired, but also Ephb4 the receptor for Ephrin B2, is underexpressed, and therefore, angiogenesis does not occur normally [28] (Figure 4).

‘ErbB signaling pathway’: The enrichment of this pathway was also found to occur in chronic wounds. During early chronicity, the genes for the ErbB receptors, Erbb2 and Erbb3, are 2.33 and 3.20 log_2_ fold overexpressed, respectively (Table 4). Erbb2 or Her2 is a protooncogene and is a constitutively active tyrosine kinase belonging to the epidermal growth factor receptor family. It is known to heterodimerize with other members of the ErbB family, one of them being ErbB3. ErbB3 has also been identified as a protooncogene. The overexpression of both Erbb2 and Erbb3 genes indicates an expression pattern that is common in both chronic wounds and cancer. Indeed, it has been shown that overexpression of the ErbB2 receptor in the epidermis of mice leads to hyperproliferation and spontaneous skin tumor development [39].

‘Focal adhesion’: This pathway is another enriched pathway in early chronicity, and it plays an integral role in wound repair. Cell adhesion is critical for cell proliferation and migration. Formation of focal adhesions is a closely regulated process that involves interactions of the cell receptors integrins with extracellular matrix (ECM) molecules, including laminin, a component of the basement membrane that underlies the epithelium. Functional integrin receptors are composed of two subunits: 1 alpha and 1 beta. The laminins (Lam a3, Lam b3, Lam c2) and integrins (Itg α2, α3, α6, β4, and β8) are overexpressed in chronic wounds (Table 5). Of particular importance is the finding that both components of the integrin α6β4 are overexpressed in our chronic wounds. Overexpression of α6β4 indicates improper angiogenesis in diabetic chronic wounds which contributes to impaired healing [40]. There is also evidence of crosstalk between integrins and growth factors. This interaction helps retain growth factors that are required for wound healing. For example, TGFβ is important for blood vessel maturation, a process required for angiogenesis [41,42]. In non-chronic wounds, TGFβ binds to the ECM via αvβ6 integrin and becomes activated. However, in chronic wounds TGFβ is underexpressed and the integrins are not able to bring about the activation by TGFβ in sufficient levels to cause proper healing.

‘Proteoglycans in cancer’: This pathway is associated with ECM molecules called proteoglycans. Proteoglycans function as supportive matrix molecules that direct spatial, temporal, and contextual information to cells. Syndecans (SDCs), are a type of proteoglycan that facilitate interactions of cells with growth factors such as FGF, VEGF and TGFβ that are involved in acute wound healing, particularly in vascularization. SDC1 also plays an important role in keratinocyte migration by interacting with integrins. During human keratinocyte migration, cells of the basal layer deposit an oriented matrix of laminin 332. Also, α6β4 and α3β1 integrins, expressed in the basal surface of keratinocytes, bind to laminin 332 (also known as laminin-5) and mediate their anchorage to the basal lamina. Keratinocyte migration requires phosphorylation of the β4 cytoplasmic domain by ErbB2/HER2 and engaging of the β4 and the SDC1 cytoplasmic domains [43]. In HaCat cells treated with Sdc1 siRNA, HER2 dependent migration was decreased by 90%, thereby showing that Sdc1 is essential for HER2-dependent keratinocyte migration required for wound closure [44]. Sdc1 is 1.92 log_2_ fold upregulated in our chronic wounds. In various types of cancer, Sdc1 deregulation has been linked to poor prognosis. For example, in malignant melanoma, treatment with an antibody–drug conjugate that targets Sdc1, showed anti-tumor effects by inhibiting vascular maturation [45,46]. These observations suggest that elevated Sdc1 in chronic wounds could be a marker on non-healing.

***A.3. The significantly enriched pathways identified in the brown module are***: ‘Cardiac muscle contraction’, ‘Adrenergic signaling in cardiomyocytes’, ‘Hypertrophic cardiomyopathy’, ‘Dilated cardiomyopathy’ and ‘Arrhythmogenic right ventricular cardiomyopathy’ (Table 1). All these pathways are related to cardiac muscle or cardiomyocytes. It has been shown that cardiac myofibroblasts have similar function to fibroblast that mediate wound healing in skin [47]. Both express angiogenic cytokines and α-smooth muscle actin (αSMA/Acta1 gene). Therefore, the overexpression of genes that activate fibroblasts in cutaneous wound healing are represented as pathways related to cardiac events [48,49].

**Figure 5:**
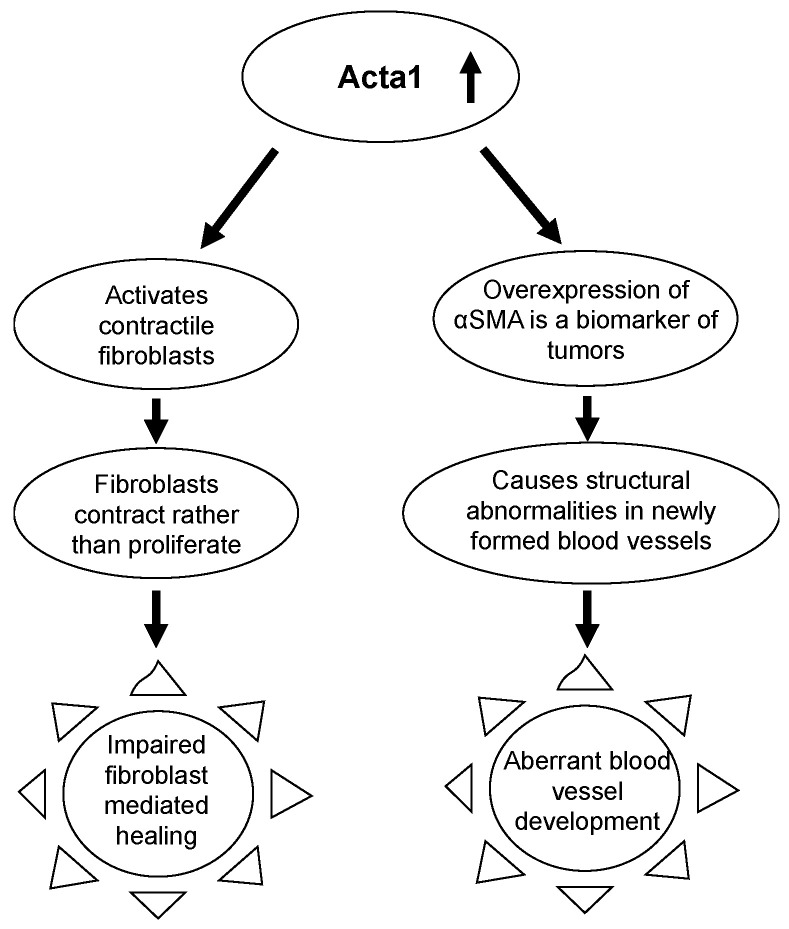
Overexpression of alpha smooth muscle actin during early chronicity. Acta1, the gene encoding for alpha smooth muscle actin is overexpressed in chronic wounds. The overexpression leads to the activation of contractile fibroblasts when healing at this stage requires fibroblast proliferation and not contractile fibroblasts. Hence fibroblast mediated healing is impaired. Overexpression of Acta1 also serves as a biomarker for tumors and is linked to causing abnormalities during neo-angiogenesis in tumors. This could indicate that Acta1 overexpression causes aberrant blood vessel development.

During wound healing fibroblasts are activated and accumulate αSMA while differentiating into myofibroblast. The contractile movements of myofibroblast are essential for wound healing during the repair phase (Figure 5) [49]. In addition to playing an important role in the differentiation of fibroblasts to myofibroblasts, expression of αSMA is a highly regulated process and is required for maturation of blood vessels. Over or under expression of αSMA causes structural abnormalities in newly formed blood vessels. In tumors which undergo neo-angiogenesis, αSMA is overexpressed [50,51]. The Acta1 gene is overexpressed in early chronicity leading to aberrant blood vessel formation similar to neo-angiogenesis in tumors. Blood vessels carry nutrients, oxygen and growth factors to the wound healing site and impaired angiogenesis therefore contributes to impaired healing (Figure 5).

### 3.2. Signaling Pathways Present in Full Chronicity

Using the RNAseq data for WGCNA when the wounds are fully chronic, we found that the genes in the grey60 (0.72, *p* = 0.002) and red (0.67, *p* = 0.006) modules correlated significantly with full chronicity (Figure 1B). The grey60 module has 154 genes and red module has 500 genes. Because the non-chronic wounds are just about healed at 20 days, we compared the log fold change of the genes in these modules at full chronicity (wounds are fully chronic at 20 days post-wounding) with those at early chronicity to identify differences in wound progression to chronicity (Tables S6–S8). Under these conditions, the genes in the red module showed no significant enrichment of pathways. However, in the grey60 module, the significantly enriched pathways are ‘JAK-STAT signaling pathway’, ‘Neuroactive ligand-receptor interaction’, ‘Wnt signaling pathway’, ‘Pathways in cancer’ and ‘Melanoma’, (Table 6).

**Figure 6:**
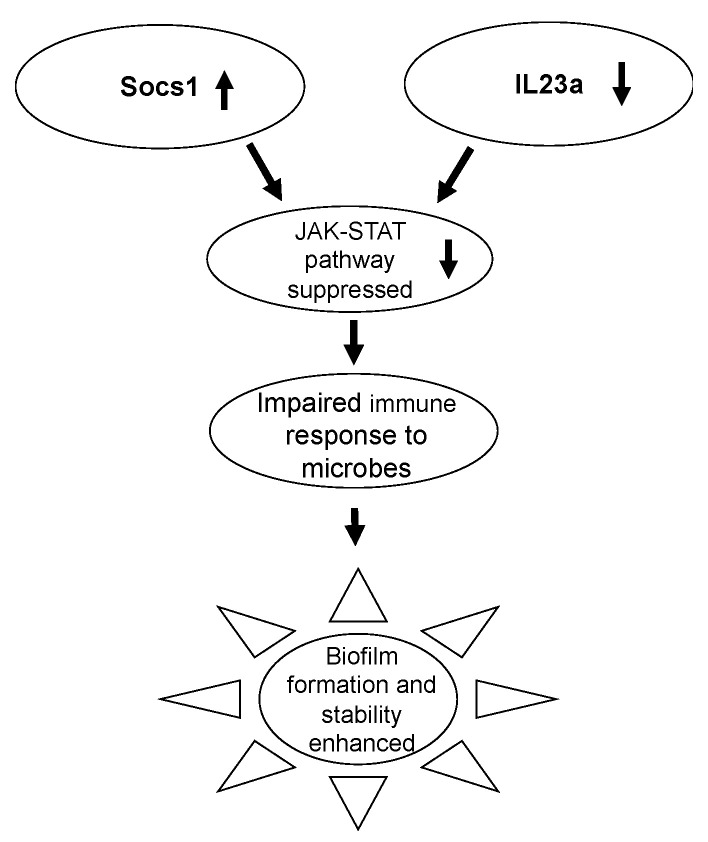
Implications of inhibition of JAK–STAT Pathway in full chronicity. Socs1 is a negative regulator of the JAK–STAT pathway; therefore, its overexpression downregulates JAK–STAT, as is shown by the underexpression of STAT genes. As an additional level of downregulation, Il23a is also underexpressed in chronic wounds. The IL23 heterodimeric cytokine complex which forms when IL23a binds to IL23r activates STAT3. Phosphorylated STAT3 then dimerizes and translocates to the nucleus where it turns on gene expression associated with adaptive and innate immunity. This leads to the stimulation of type 17 helper T (Th17) cells, which are crucial for host defense response against invading microbes and fungi. Downregulation of Il23a shows that JAK–STAT is downregulated, and therefore, stimulation of Th17 is downregulated as well, which could contribute to microbes persisting in chronic wounds leading to biofilm formation.

‘JAK–STAT signaling pathway’: This pathway involves the activation of the STAT transcription factors which bind to DNA and stimulate the transcription of genes involved in hematopoiesis of immune cells and in the inflammatory response [52]. The Socs1 (suppressor of cytokine signaling) gene is a master regulator of the JAK–STAT signaling pathway. It has been shown that it inhibits JAK1 and JAK2 through its kinase inhibitory region, which binds to the substrate binding groove of JAK with high specificity, thereby inhibiting JAK1 and JAK2 function [53]. Jak1 and Jak2 are both underexpressed while Socs1 is overexpressed in fully chronic wounds (Figure 6). This indicates that the JAK–STAT pathway is suppressed in chronic wounds, both because Jak1 and Jak2 are underexpressed and because, when present, they are inhibited by Socs1.

The IL23 heterodimeric cytokine complex which forms when IL23a binds to IL23R, can also activate the JAK–STAT pathway via phosphorylation of JAK2 and STAT3. Phosphorylated STAT3 then dimerizes and translocates to the nucleus where the complex binds to promoter regions for genes that control adaptive and innate immunity. This leads to the stimulation of type 17 helper T (Th17) cells, which are crucial for host defense response against invading microbes [54,55,56]. In chronic wounds, IL23a is under-expressed during full chronicity, which shows that the JAK–STAT pathway is impaired and Th17 cells are not stimulated to fight infections in chronic wounds (Figure 6).

‘Neuroactive ligand receptor interaction’: this pathway contains genes such as Adra2a, which is upregulated. As mentioned above the Adra2 gene mediates catecholamine-induced inhibition of adenylate cyclase which reduces cAMP production and adversely affects keratinocyte migration [57]. We found that during early chronicity, cAMP production is adversely affected by the Adra2b gene whereas during full chronicity, it is Adra2a that is overexpressed. The other gene involved in enrichment of ‘neuroactive ligand receptor interaction’ was a receptor for bradykinin (Bdkrb1). BDKRB1 is a G-protein coupled receptor which binds bradykinin, a peptide that promotes inflammation. Bdkrb1 is also expressed in basal and suprabasal keratinocytes, and its overexpression could cause dysregulation of keratinocyte proliferation causing epidermal thickening by overproduction of keratinocytes at the wound edge [58]. Therefore, deviation from normal in these pathways affects re-epithelialization by both inhibiting keratinocyte migration and inducing proliferation at the wound edge.

**Figure 7:**
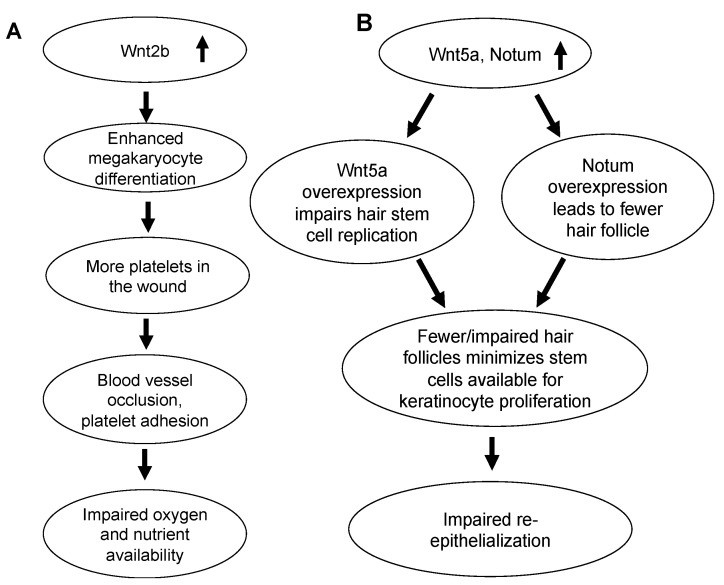
Wnt signaling is dysregulated in fully chronic wounds. During full chronicity, Wnt2b, Wnt5a, and Notum are overexpressed. (**A**) Wnt2b overexpression is seen during differentiation of megakaryocytes that give rise to platelets. Excess platelets during full chronicity may cause blood vessel occlusion and platelet adhesion and blocks oxygen and nutrients from reaching the wound. (**B**) Overexpression of Wnt5a and Notum impairs hair stem cell replication and causes fewer hair follicles to form, respectively. Hair follicles provide stem cells for keratinocyte proliferation; formation of fewer follicles therefore impairs keratinocyte proliferation, thereby affecting re-epithelialization.

‘Wnt signaling pathway: Genes involved in this pathway are Wnt2b, Wnt5a, and Notum. In chronic wounds, Wnt2b is upregulated 1.95-fold, Wnt5a 2.99 fold and Notum 3.29 fold. APC and β-catenin expression levels are similar in chronic and non-chronic wounds. Wnt2b upregulation in K562 immortalized lymphoblast cell lines causes differentiation to megakaryocytes. Megakaryocytes give rise to platelets [59]. In TNFSF14/LIGHT^−/−^ knockout mice, which have impaired healing, high levels of platelets were observed in the wound. Such accumulation of platelets contributes to blood vessel occlusion and enhances platelet adhesion which contributes to impaired healing by depriving the wound of oxygen and nutrients [60] (Figure 7A). Wnt5a overexpression impairs hair follicle anagen, which is the phase in which the hair stem cells are actively replicating. This hair follicle process in skin promotes wound healing by providing stem cells for keratinocyte proliferation [61,62]. Overexpression of Notum is also known to cause significantly fewer hair follicle in transgenic mice (Krt14rtTA) [63]. Therefore, overexpression of Wnt5a and Notum reduces the number of hair follicle in anagen, which minimizes the stem cells available for keratinocyte proliferation. Lack of keratinocyte proliferation in turn negatively impacts the re-epithelialization process in chronic wounds, which ultimately impairs wound-healing (Figure 7B).

‘Pathways in cancer’ ‘and ‘Melanoma’: The enhanced presence of these pathways at full chronicity strongly suggests again that chronic wounds exhibit processes like cancer. Some of the genes involved in both ‘Pathways in cancer’ and ‘Melanoma’ are Fgf2, Fgf5, and Pdgfa. All three genes are overexpressed during full chronicity. Fgf2 accelerates epithelial to mesenchymal transition (EMT) in keratinocytes, a process that is known to occur during cancer development [64]. Fgf2 in acute wound-healing is also a pro-angiogenic factor. However, at full chronicity, other genes such as Fgf-bp1 are also overexpressed. Tumor growth and progression is greatly aided by the expression of Fgf-bp1, which act as an FGF chaperone and protects FGF against proteolytic degradation. The overexpression of Fgf-bp1 shows correlation with a high density of microvessel development, which is known as a marker for malignancy [65]. Fgf5 functions as an inhibitor of hair elongation during the anagen IV phase of hair growth [66]. Overexpression of Fgf5, therefore, causes impaired anagen. It is during this phase that the hair follicle provides stem cells for keratinocyte proliferation [67,68]. Therefore, overexpression of Fgf5 adversely affects keratinocyte proliferation. Sustained Pdgfa overexpression has been linked to the cancer [69,70]. Therefore, the overexpression of these mitogens during full chronicity is detrimental for wound-healing as it dysregulates healing processes much as they do in tumor development.

## 4. Discussion

Previous research from our laboratory used a systems biology approach to analyze Nanostring data using WGCNA to identify signaling pathways during the first 48 hrs post-wounding in chronic wounds. Our current paper focusses on the progression of diabetic wounds towards full chronicity. All pathways are in many ways the result of the increased oxidative stress caused by ROS in the first 24 h after wounding. Much like with the RNAseq approach presented here, our previous studies predicted an upregulation of catecholamine production in chronic wounds from very early on in response to injury. Catecholamines (dopamine, epinephrine, and nor-epinephrine) adversely affect keratinocyte migration during re-epithelialization [71,72]. Overexpression of epinephrine prevents re-epithelialization which affects wound closure. This problem continues during early chronicity because the ADRA2 adrenergic receptor is overexpressed in chronic wounds. ADRA2 mediates catecholamine-induced inhibition of adenylate cyclase, which is responsible for production of cAMP, a second messenger that promotes actin cytoskeleton remodeling and cell migration [73]. Interestingly, during early chronicity, cAMP production is adversely affected by the Adra2b gene whereas during full chronicity it is the Adra2a gene that is overexpressed. These are two common functional polymorphisms of the alpha2-adrenergic receptor, and both can mediate physiological effects of epinephrine and nor-epinephrine [74]. Whether these two functional polymorphisms are interchangeable in chronic wounds, or whether they are specific to the stage of chronicity, is not known.

It has been shown that, application of DBcAMP (an analog of cAMP) to wounds, results in significantly higher re-epithelialization and granulation tissue formation when compared to saline-treated control wounds [26]. Therefore, the inhibition of cAMP producing enzymes by catecholamines severely impairs wound healing strongly suggesting that treatments to reverse this inhibition could be very effective in stimulating re-epithelialization and formation of the healing tissue.

Proteoglycans are components of the extracellular matrix. Sdcs (heparan sulfate proteoglycans), are a type of proteoglycans that facilitate interactions of cells with growth factors such as FGF, VEGF, and TGFβ, which bind to the heparan sulfate chain on the proteoglycans. Sdc1 is expressed in keratinocytes, fibroblasts, and epithelial cells and it has been known to play a role in wound-healing by activating integrins which in turn facilitate cell migration and ECM assembly [75]. Sdc1 is also important in the formation and disassembly of focal adhesion during migration of epithelial cells. Overexpression of Sdc1 is linked to basal and squamous cell carcinomas, a metastatic type of human skin cancer. During the spread of cancer cells, proteoglycans on metastatic cells or their target tissues control tumor cell movement and dissemination patterns [76]. Sdc1 is overexpressed during early chronicity and therefore indicates increased tumor-like mobility in keratinocytes, reminiscent of melanomas.

Focal adhesion proteins such as integrins and laminins are important for keratinocyte migration [77,78,79]. In the RNAseq data presented here, several laminins and integrins are overexpressed in chronic wounds. For example, both components of integrin α6β4 are overexpressed. This integrin is a receptor for laminin 332 (also known as laminin 5), which forms hemidesmosomes in keratinocytes. In normal skin, α6β4 is expressed in the basal layer of keratinocytes and mediates cellular adhesion to the basement membrane. During normal wound-healing, as the keratinocytes need to migrate to cover the open wound area, the integrins expressed on the keratinocytes switch from α6β4 to α3β1 integrin which then binds to laminin 5 (on the basement membrane) disassembling the hemidesmosomes enabling keratinocyte migration. Keratinocyte migration is also increased by cytokines such as Il-1, Il-6, and 1l-17 [80]. However, in our chronic wounds data, although integrin α3 is overexpressed, Itgβ1, Il-1, Il-6, and Il-17 are all downregulated indicating that the disassembly of the hemidesmosomes for keratinocytes to cover the wound does not occur as it would in normal wound-healing. Therefore, dysregulation of the α6β4 disrupts epidermal homeostasis [81].

One of the most important functions of α6β4 integrin is to bind Lam b3, which is the β-subunit of Laminin 5. Our data shows overexpression of α6β4 and Lam b3 during early chronicity. The integrin α6β4 has been identified as one of the central regulators of tumor angiogenesis. This integrin is only expressed in mature blood vessels and not developing blood vessels. Deletion of the 1355 threonine on the substrate domain of β4 significantly inhibited angiogenesis in 80% of tested tumor xenograft models, further suggesting a crucial role for α6β4 in tumor angiogenesis [82]. Therefore, the overexpression of α6β4 in early chronicity suggests tumorigenic angiogenesis leading to defective blood vessel formation.

During early chronicity, fibroblast-mediated tissue repair takes place. It has been shown that Acta 1 (the gene for αSMA) overexpression leads to poor proliferation, thereby impairing wound-healing [83]. Our data shows that Acta1 is 2.8 log_2_ fold overexpressed in chronic wounds, which indicates that at the stage when fibroblast mediated repair should be occurring, their proliferation is hampered by Acta1 overexpression.

In normal wound-healing, the JAK–STAT pathway activates the STAT family of transcription factors, which in turn impact the transcription of genes that regulate recruitment, survival, and activation of immune cells [84]. Our data show that at full chronicity, the JAK–STAT pathway is suppressed. The overexpression of Socs1 and the underexpression of Il23a both indicate that the JAK–STAT pathway is inactive in chronic wounds. The most significant downstream effect of this inactivation is that Th17 cells are not stimulated. Th17 cells are required to mount a host defense response and fight microbial infection. Chronic wounds show microbial invasion as early as 24 hrs after injury and by full chronicity, many chronic wounds sustain antibiotic resistant biofilm. The unavailability of mature Th17 cells can explain why the host immune response does not engage in fighting biofilm in chronic wounds.

## 5. Summary and Conclusions

**Figure 8:**
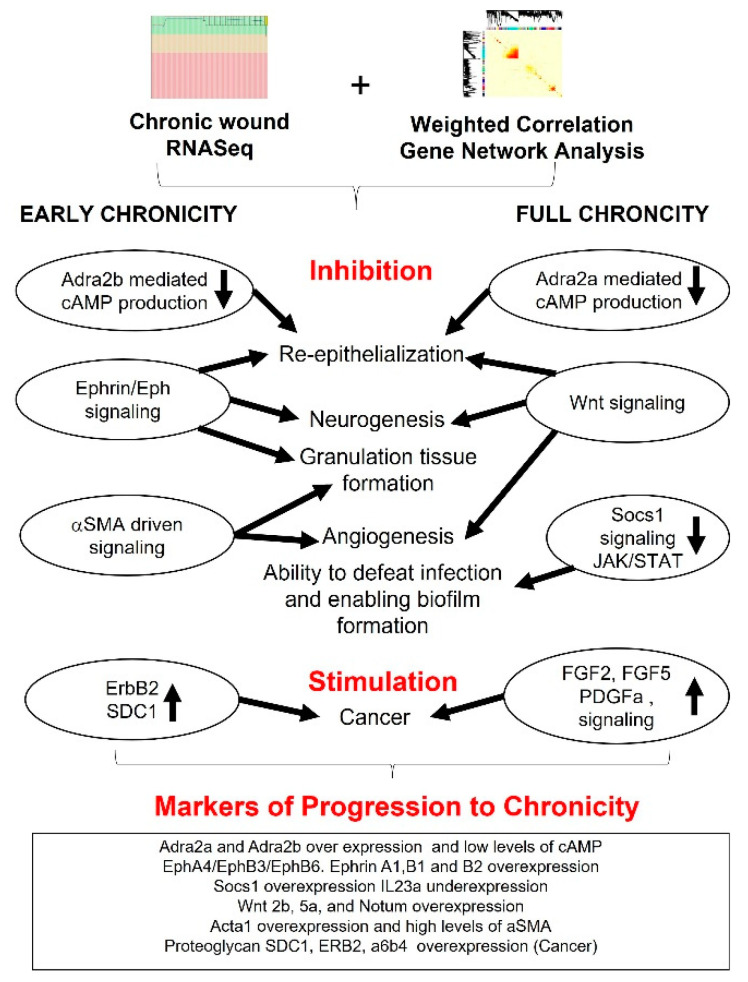
Progression to wound chronicity. For this study, we isolated mRNA extracted from chronic and non-chronic tissue. RNASeq was completed followed by analysis using WGCNA. WGCNA identified significantly correlated gene modules (*p* < 0.05), which yielded significantly enriched (FDR < 0.05) signaling pathways in early and full chronicity. During early chronicity, Adra2b mediated lowering of cAMP production (impairs re-epithelialization and signal amplification), Ephrin/Eph signaling (impairs re-epithelialization, neurogenesis, and granulation tissue formation), aSMA-driven signaling (impairs granulation tissue formation and angiogenesis) were enriched. While in full chronicity, Adra2a mediated lowering of cAMP production, Wnt signaling (impairs re-epithelialization, granulation tissue formation and angiogenesis). Socs1 was upregulated which suppresses JAK–STAT signaling (subduing host immunity and enabling biofilm formation). In addition, overexpression of ErbB2/HER2 and SDC1 in early chronicity and Fgf2, Fgf5, and Pdgfa overexpression in full chronicity promote cancer-like processes.

In this study, we found the dysregulation of key wound-healing signaling pathways that can potentially lead to progression of a wound to chronicity (Figure 8). One of these pathways that is dysfunctional from early to full chronicity is the catecholamine driven signaling. When epinephrine interacts with ADRA2, a β-adrenergic receptor in keratinocytes, it inhibits adenylate cyclase which in turn lowers the production of cAMP. The deficiency of cAMP impairs re-epithelialization and granulation tissue formation, two hallmarks of chronic wounds. Because treatment with the cAMP analog, DBcAMP, improves re-epithelialization and granulation tissue formation, it is possible that treatment with cAMP analogs could prove to be a fruitful way of treatment. It is also possible that cAMP levels in chronic wounds can be increased by inhibiting cAMP-phosphodiesterase (PDE), an enzyme that degrades cAMP [85].

Another major pathway that is dysfunctional in chronic wounds during early chronicity is the “axon guidance”. The Ephrins/Eph-induced signaling is associated with growth cone collapse that impairs motor nerve regeneration which in turn adversely affects sensory nerve regeneration. Because several Ephrins/Eph are overexpressed growth cone regeneration does not occur in chronic wounds. The Ephrin/Eph pathways are also important for re-epithelialization as well as granulation tissue formation and angiogenesis.

Significant modules at full chronicity showed the suppression of JAK–STAT pathway by Socs1 and IL23a, which explains lower immunity and defense against bacterial pathogens colonizing the wound, thus aiding biofilm formation. In addition, genes overexpressed in the ‘Wnt signaling pathway are associated with maintaining a pro-inflammatory state leading to skin disorders, such as psoriasis, as well as affecting the production of hair follicle stem cells that are critical for re-epithelialization.

Moreover, during early chronicity, the signaling pathways involving proteoglycans are also dysfunctional. Sdc1 in chronic wounds autoactivates ErbB2, which is a characteristic of melanoma, suggesting that as wounds become chronic, they show many characteristics of cancer. In addition, when the integrin α_6_β_4_ is overexpressed, blood vessels develop, which is characteristic of tumors. Indeed, similarities in gene expression between tumors and chronic wounds was first introduced by Harold Dvorak in a seminal paper [86]. Therefore, Sdc1, ErbB2 and α_6_β_4_ can be potentially targeted to treat chronic wounds with drugs that are already on the market.

Our findings identify signaling pathways and potential targets that can be manipulated to coax a chronic wound to heal and potentially help in the development of new treatments for chronic wounds.

## Figures and Tables

**Table 1:** Significant pathways in early chronicity.

Module in WGCNA	Pathway Description	Fold Enrichment (FDR < 0.05)
Turquoise	Axon guidance	1.95
	ErbB signaling pathway	1.21
	Proteoglycans in cancer	1.37
	Regulation of actin cytoskeleton	1.15
	Focal adhesion	1.05
Cyan	Neuroactive ligand-receptor interaction	1.1
Brown	Cardiac muscle contraction	10.38
	Dilated cardiomyopathy	7.07
	Hypertrophic cardiomyopathy	6.53
	Adrenergic signaling in cardiomyocytes	5.14
	Arrhythmogenic right ventricular cardiomyopathy	2.73

**Table 2:** Expression of ephrinA and its receptors, in chronic wounds.

Gene Name	Description	cday1-ncday1_logFC
Efna1	Ephrin A1	1.30
Efna2	Ephrin A2	0.84
Efna3	Ephrin A3	4.34
Efna4	Ephrin A4	2.67
Efna5	Ephrin A5	2.03
Epha1	Eph receptor A1	3.33
Epha2	Eph receptor A2	2.32
Epha4	Eph receptor A4	3.13
Epha5	Eph receptor A5	−2.2
Epha7	Eph receptor A7	2.44

**Table 3:** Expression of ephrinB and its receptors in chronic wounds.

Gene Name	Description	cday1-ncday1_logFC
Efnb1	Ephrin B1	1.92
Efnb2	Ephrin B2	2.61
Efnb3	Ephrin B3	1.24
Ephb2	Eph receptor B2	1.09
Ephb3	Eph receptor B3	2.23
Ephb4	Eph receptor B4	0.8
Ephb6	Eph receptor B6	3.33

**Table 4:** Expression of Erbb receptors in chronic wounds.

Gene Name	Description	cday1-ncday1_logFC
Erbb2	erb-b2 receptor tyrosine kinase 2	2.33
Erbb3	erb-b2 receptor tyrosine kinase 3	3.21

**Table 5:** Expression of laminins and integrins in chronic wounds.

Gene Name	Description	cday1-ncday1_logFC
Itga2	integrin alpha 2	3.79
Itga3	integrin alpha 3	1.9
Itga4	integrin alpha 4	−1.33
Itga6	integrin alpha 6	1.95
Itgb1	integrin beta 1	−0.34
Itgb4	integrin beta 4	2.63
Itgb8	integrin beta 8	2.67
Lama3	laminin, alpha 3	4.24
Lamb3	laminin, beta 3	3.26
Lamc2	laminin, gamma 2	3.53

**Table 6:** Significant pathways in full chronicity.

Module in WGCNA	Pathway Description	Fold Enrichment
Grey60	JAK-STAT signaling pathway	4.76
	Neuroactive ligand-receptor interaction	3.09
	Wnt signaling pathway	3.94
	Pathways in cancer	2.90
	Melanoma	5.39

## Data Availability

Data is contained within the article or supplementary material.

## Supplementary Materials

The following supporting information can be downloaded at: https://www.mdpi.com/article/10.3390/antiox11081506/s1, The genes included in each module along with their gene trait significance and module membership significance are provided as supplementary materials. Table S1. Raw counts for genes identified in RNASeq. Table S2. TPM normalized counts for genes identified in RNASeq. Table S3. Genes in cyan module significantly correlated to early chronicity. Table S4. Genes in turquoise module significantly correlated to early chronicity. Table S5. Genes in brown module significantly correlated to early chronicity. Table S6. Genes in grey60 module significantly correlated to full chronicity (Day20 vs. Day1). Table S7. Genes in grey60 module significantly correlated to full chronicity (Day20 vs. Day5). Table S8. Genes in grey60 module significantly correlated to full chronicity (Day20 vs. Day10).
